# Beyond the acute: pain in long COVID survivors at 1.5 years

**DOI:** 10.1007/s10072-024-07620-7

**Published:** 2024-05-31

**Authors:** Emel Oguz-Akarsu, Gizem Gullu, Erhan Kilic, Yasemin Dinç, Gonul Akdag, Cansu Rehber, Necdet Karli

**Affiliations:** 1 Department of Neurology, Uludag School of Medicine, Bursa Uludag University, Gorukle, Bursa, Turkey; 2 Department of Neurology, Faculty of Medicine, Kutahya Health Sciences University, Kutahya, Turkey

**Keywords:** Pain, Headache, Myalgia, Neuropathic pain symptom, long-COVID

## Abstract

**Objective:**

Long COVID, characterized by persistent symptoms post-acute COVID-19, remains a subject of intense investigation. This study focuses on pain, a common and notable symptom reported by long COVID patients.

**Method:**

A cohort of 191 individuals, initially diagnosed with mild-to-moderate COVID-19, was followed up 1.5 years later to assess the frequency, clinical characteristics, and factors associated with pain persistence.

**Results:**

Our study revealed that 31.9% of participants experienced at least one persistent pain symptom after 1.5 years. Headache emerged as the most prevalent symptom (29.8%), followed by myalgia (5.8%) and neuropathic pain (4.2%). Factors such as female gender and the presence of neuropathic pain symptom were identified as predictors of long-term headaches. Myalgia, showed associations with headache, arthralgia, and low ferritin levels. Persistent neuropathic pain symptom (4.2%) was linked to older age, female gender, sore throat, and headache.

**Conclusion:**

This study provides insights into the evolution of pain symptoms over time after COVID-19 infection, emphasizing the interconnection between different pain syndromes. This research contributes to understanding the diverse and evolving nature of pain in long COVID survivors, offering valuable insights for targeted interventions and further investigations into the underlying mechanisms of persistent pain.

**Supplementary Information:**

The online version contains supplementary material available at 10.1007/s10072-024-07620-7.

## Key messages

Our study showed that nearly 32% of participants experienced at least one type of pain after 1.5 years of mild-to-moderate COVID-19 infection. This study provides insights into the evolution of pain symptoms over time, emphasizing the interconnection between different pain syndromes in long COVID survivors.

**Supplementary Information:**

The online version contains supplementary material available at 10.1007/s10072-024-07620-7.

## Introduction

The COVID-19 pandemic has cast a long shadow, with the acute phase of infection often followed by a complex and persistent condition known as long COVID. Defined by a broad spectrum of symptoms that linger beyond the initial illness, long COVID significantly impacts the lives of millions worldwide [1]. This heterogeneous condition encompasses over 50 overlapping symptoms, with pain emerging as a prevalent and concerning feature [2]. Pain in long COVID manifests in various forms, including headache, myalgia, joint pain, and neuropathic pain [3, 4]. A UK investigation of over 270,000 individuals revealed that 5.71% reported chest/throat pain, 4.63% experienced headaches, and an additional 7.19% had other types of pain within 3 to 6 months post-infection [5]. Similarly, research from Wuhan, China, reported a prevalence of headache and myalgia in 2.3% and 7.9% of COVID-19 survivors, respectively, one year after hospital discharge [6].

The definition of long COVID, and consequently its estimated prevalence and long-term outcomes, remains under debate. Existing studies offer divergent findings, with one prospective study reporting a 17% failure to recover after 24 months [7], while another demonstrates an 8% recovery rate over 23 months[8]. Notably, a meta-analysis suggests that approximately 15% of patients continue to experience long COVID symptoms one year after infection [9]. Furthermore, over half of patients referred to a post-COVID clinic exhibited no improvement 1.5 years after the initial infection [10]. To address these discrepancies and gain a better understanding of long COVID’s trajectory and potential long-term pain, we conducted a follow-up study at the 1.5-year mark.

While the exact causes of long COVID remain under investigation, several theories have been proposed. These include the prolonged presence of the virus, reinfection with different SARS-CoV-2 strains, endothelial damage, excessive blood clotting, dysfunctional immune response, and chronic inflammation [3]. Similarly, the risk factors associated with developing post-COVID symptoms are not fully elucidated. While some studies highlight female sex and older age as contributing factors, others emphasize the potential role of psychological factors [11, 12]. A recent nationwide survey on post-COVID musculoskeletal pain revealed that older age, female sex, higher BMI, and a pre-existing medical history encompassing conditions like migraine, whiplash, and type-2 diabetes, as well as lower socioeconomic status, are potential risk factors for developing new widespread post-COVID pain [13].

In our preceding study focused on COVID-19 and pain, we investigated the frequency, clinical characteristics, and associated factors of pain syndromes in mild-to-moderate COVID-19 patients [14]. This present study represents an extension of our prior study, thoroughly investigating the long-term assessment of pain persistence in long COVID survivors at the 1.5-year mark.

## Method

During the pandemic of COVID-19, we conducted a study in Bursa, a city in northwestern Turkey. The primary objective of this study was to assess the frequency, clinical characteristics, and related factors of pain syndromes in mild-to-moderate COVID-19 patients. The study included patients aged 18 and older who had been diagnosed with COVID-19 through reverse transcription-polymerase chain reaction (RT-PCR) using oropharyngeal and nasal swab samples. Patients were contacted by phone and interviews took place 1.5–3 months after a positive PCR test, excluding those with severe illness, cognitive issues, and unreliable responses. Two experienced neurologists interviewed patients who agreed to participate in the study [14].

Participants initially participated in structured interviews to collect data on demographic information, COVID-19 diagnosis and treatment, vaccination status, pre-existing medical conditions, and any previous chronic pain. During these interviews, individuals were questioned about the development of new-onset pain during their SARS-CoV-2 infection, including its location, duration, and characteristics.

Patients were asked if they experienced headaches during their COVID-19 infection, and their characteristics were recorded. Pre-existing headaches were classified according to ICHD-3 criteria. Patients were also asked about new-onset myalgia, neuropathic pain, and polyarthralgia. New-onset neuropathic pain during the acute phase of COVID-19 was defined as neuropathic symptoms occurring within 30 days of the diagnosis. It included symptoms like tingling, burning, and electric shocks localized to specific areas. Computed tomography of the thorax and various laboratory tests were assessed from patient records. Details of this initial survey and sampling methodology are described elsewhere [14].

After 1.5 years of the first study, we conducted a follow-up telephone survey, specifically targeting participants who had reported pain in the initial study. To ensure the accuracy of the results, we made multiple attempts, up to three in total, to reach each patient who had reported pain in the initial study within a one-month timeframe. The follow-up telephone interviews were conducted by two researchers, (whom participated in the first study) who collectively assessed each subject using a standardized protocol.

The second telephone survey included a 37-item questionnaire designed for diagnosing persistent pain syndromes and delineating their characteristics. Each telephone survey was conducted within a duration of 20–25 min. Our assessment focused on the evolution of pain experiences between the initial and second assessments after 1.5 years, particularly focusing on newly reported pain. Headache was an exception due to its high prevalence in the general population. Therefore, we also analyzed changes in headache characteristics, including the worsening of existing headaches and the emergence of new headache types.

We used semi-structured questionnaires designed to diagnose persistent pain syndromes after COVID-19 infection, delineate their characteristics, and assess treatment experiences. It covered various types of persistent pain, including persistent headache (location, duration, severity, associated symptoms, treatment), persistent myalgia (localization, severity, treatment), persistent polyarthralgia (severity, treatment), persistent neuropathic pain was assessed using the Neuropathic Pain Questionnaire (NPQ) (**Turkish version validated** ) [15, 16] (suppl). The questionnaire’s design allowed for a longitudinal assessment of participants’ pain experiences by following up on initial questions, enabling us to identify both new-onset and persistent pain.

Commencing with an initial cohort of 222 patients, the subsequent study included 191 individuals, resulting in a response rate of 86%. Subsequently, we evaluated the factors associated with persistent pain syndromes. We conducted comparative analyses between patients manifesting persistent symptoms—such as headache, myalgia, neuropathic pain, and arthralgia—and those presenting with non-persistent manifestations in these specific categories.

Verbal informed consent was obtained from each participant before their inclusion into the study. The ethical committee approved the study protocol.

Continuous variables were compared between groups using independent sample t-tests or the Mann–Whitney U test. Categorical variables were analyzed for differences using two-sided Fisher’s exact, Pearson’s chi-square, and continuity correction tests. Binary logistic regression tests were used to identify variables that differentiate between patient groups, using the backward regression variable selection method. The logistic regression analysis included variables with p-values less than 0.05 in the univariate analysis. All statistical analyses were conducted using IBM SPSS Statistics Version 21.0. Statistical significance was determined at *p* ≤ 0.05.

## Results

### Pain in patients with long COVID

**Fig. 1:**
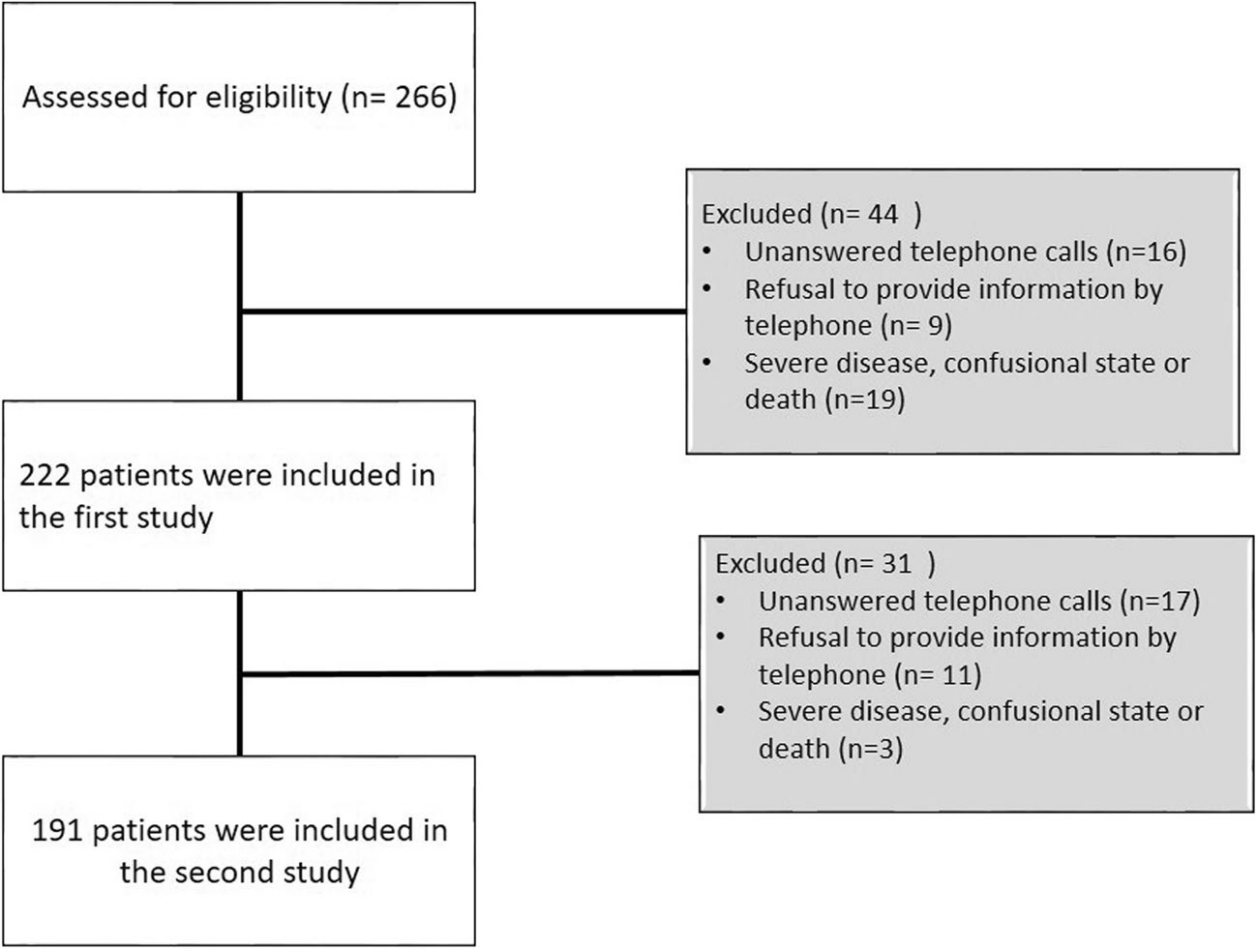
Flowchart of the patients included into the study

The primary findings of our first study focusing on pain in the acute phase of COVID-19 have been previously published. The present study focused on long COVID pain symptoms after 1.5 years of COVID-19 infection. During the follow-up period, 191 participants were included in the final study, as illustrated in the flowchart provided (Fig. 1).

Table 1 presents baseline characteristics of 191 patients with SARS-CoV-2 infection. Out of the 191 patients, 92 were females (48.2%) and 99 were males (51.8%). The average age of the patients was 41.4 ± 13.7 years. About 30.9% of the 191 patients had comorbidities such as hypertension, cardiovascular disease, diabetes mellitus, and others.

**Table 1:** Baseline characteristics of 191 patients with SARS-CoV-2 infection

	All patients (191 patients)
Age (years) (minimum-maximum)	41.4 ± 13.7 (18–87)
Sex	
Female	92 (48.2%)
Current smoker	30 (15.7%)
Comorbidities	59 (30.9%)
Number of immune compromised patients	6 (3.1%)
Number of hospitalized patients	120 (62.8%)
Number of patients placed in intensive care unit	3 (1.6%)
Number of patients with COVID-19 pneumonia	93 (48.7%)*

*a total of 189 patients underwent chest CT

**Fig. 2:**
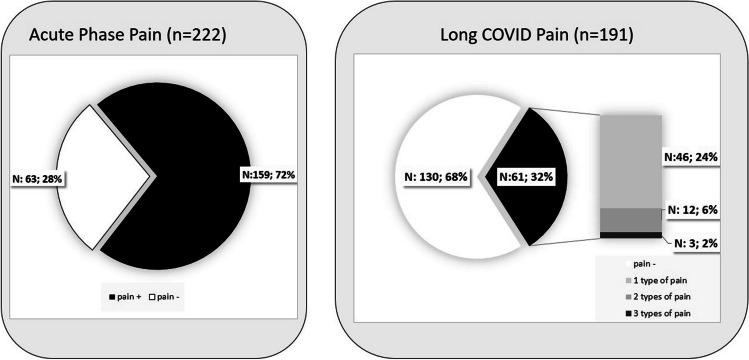
The frequency of of pain symptoms during the acute and long COVID phases

Out of the 191 patients, 31.9% (61 patients) reported persistent pain after 1.5 years of COVID-19 infection. A total of 46 patients, accounting for 24.1% of the participants, reported a single type of long COVID pain, while 12 individuals (6.3%) reported two, and 3 (1.6%) reported three distinct types of pain during the long COVID period (Fig. 2).

**Fig. 3:**
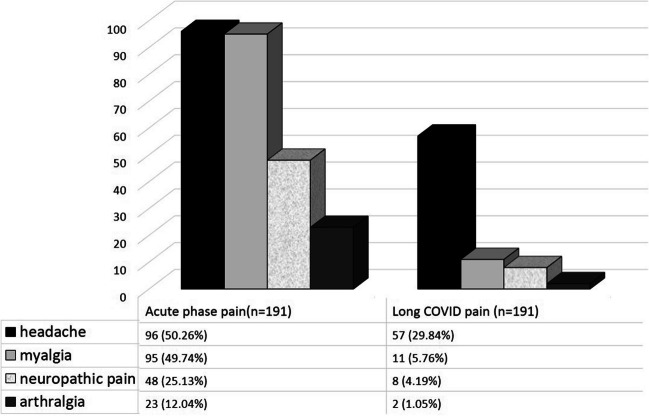
The distribution of pain symptoms during the acute and long COVID phases

**Fig. 4:**
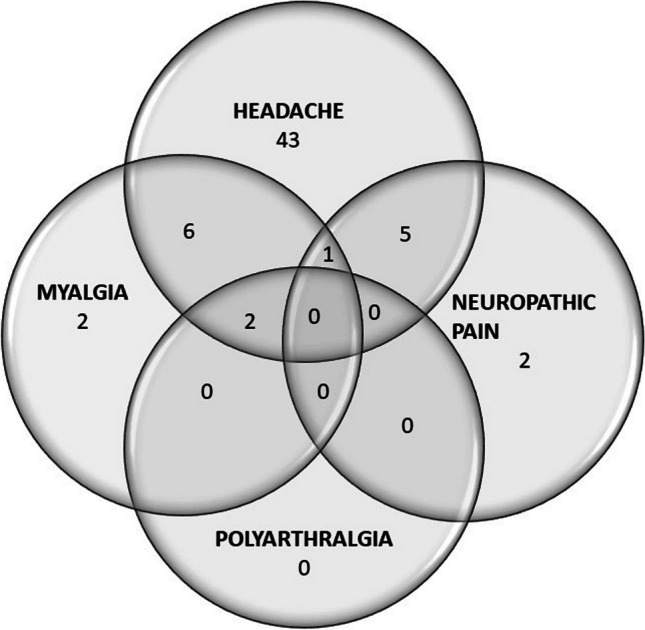
The overlap between different persistent pain syndromes Note: The numbers in the figure represent the number of patients

Headache was the most commonly reported symptom in long-COVID, with a prevalence rate of 29.8%. This was followed by myalgia at 5.8%, while neuropathic pain and arthralgia had prevalence rates of 4.2% and 1%, respectively. Figure 3 displays the distribution of pain symptoms during the acute and long COVID phases. The overlap between different persistent pain syndromes is shown in Fig. 4.

### Headache

The majority of patients experienced a bilateral headache phenotype (80.7%), characterized by a pulsating quality (47.4%) and severe or moderate intensity (70.2%). Table 2 provides a summary of the phenotype and associated symptoms. Of the 191 patients under consideration, a significant proportion of 96 patients initially presented with headaches. A subsequent examination of these cases revealed that the headache persisted in 59.37% (57) of these patients. Among the 57 participants who reported long- COVID headaches, 27 had a history of migraine, 21 had a history of tension-type headaches, and 1 had a history of other types of headaches. New-onset headaches were reported by 7 (3.66%) participants. Additionally, 39 patients reported an exacerbation of their previous headache, with 21 of them having migraine, 17 having tension-type headaches and 1 with migraine and medication overuse headache. Furthermore, 11 patients had a change in their headache type. The transition happened to be from migraine to tension-type headache and vice versa.

**Table 2:** Headache characteristics of patients with long COVID

Headache frequency	< 1 day/month	3 (5.3%)
	**1–4 days/month**	**42 (73.7%)**
	5–14 days/month	7 (12.3%)
	≥ 15 days	5 (8.8%)
Headache severity	Mild	17 (29.8%)
	**Moderate**	**19 (33.3%)**
	**Severe**	**21 (36.9%)**
Headache characteristics	**Pulsating**	**27 (47.4%)**
	Pressing	22 (38.6%)
	Fiery	2 (3.5%)
	Stabbing	3 (5.3%)
	Other	3 (5.3%)
Localization	Only unilateral	8 (14%)
	**Only bilateral**	**46 (80.7%)**
	Bilateral predominant one side	3 (5.3%)
Associated symptoms	Nausea/vomiting	23 (40.4%)
	Photophobia	16 (28.1%)
	Phonophobia	19 (33.3%)
	Aggravation with movement	25 (43.9%)

A total of 33 patients received medication to manage their headaches. These medications were primarily for acute treatment. Thirteen patients used only paracetamol, 12 used only NSAIDs, 3 used only triptans, and 5 used combination analgesics. Notably, only one patient with high headache frequency (≥ 15 days/ month) received preventive medication.

Table 3 presents a comparative analysis of patients afflicted with long-term headaches. Among the parameters investigated, it was observed that patients with long-term headaches were predominantly female and exhibited a higher incidence of asthenia, cough, sore throat, myalgia, neuropathic pain, and low ferritin levels compared to patients without a history of long-term headaches. Furthermore, the binary logistic regression model indicated that the presence of long-term headaches could be predicted by two clinical variables, namely female gender and neuropathic pain (Table 4).

**Table 3:** Comparison of patients in relation with long term myalgia and headache

	Long COVID Myalgia (+) (11)	Myalgia (-) (180)	*P* (myalgia+/-)	Long COVID Headache (+) (57)	Headache (-) (134)	*P*’ (headache+/-)
Age (years) [median (Q3-Q1)]	41 (37–45)	40 (30–51)	0.735	39 (29.5–44.5)	41 (51.5–30)	0.160
Female sex	8 (72.7%)	84 (46.7%)	0.171	41(71.9%)	51(38.1%)	**< 0.001**
Hospitalization	8 (72.7%)	112 (62.2%)	0.749	31 (54.4%)	89(66.4%)	0.115
Hospitalization in ICU	0 (0%)	3 (1.7%)	0.666	0 (0%)	3 (2.2%)	0.255
Asthenia	5 (45.5%)	48 (26.7%)	0.177	24 (42.2%)	29 (21.6%)	**0.007**
Fever	5 (45.5%)	86 (47.8%)	1.00	29 (50.9%)	62 (46.3%)	0.335
Cough	7 (63.6%)	94 (52.2%)	0.671	37 (64.9%)	64 (47.8%)	**0.022**
Sore throat	3 (27.3%)	69 (38.3%)	0.462	28 (49.1%)	44 (32.8%)	**0.050**
Anosmia	5 (45.1%)	95 (52.8%)	0.872	34(59.6%)	66 (49.3%)	0.188
Headache	9(81.8%)	48(26.7%)	**< 0.001**	-	-	**-**
Myalgia	-	-	**-**	9 (15.8%)	2 (1.5%)	**< 0.001**
Arthralgia	2 (18.2%)	0(0%)	**< 0.001**	2 (3.5%)	0 (0%)	0.088
Neuropathic pain	1 (9.1%)	7(3.9%)	0.403	6 (10.5%)	2 (1.5%)	**0.014**
Comorbid disease	5 (45.5%)	54(30.0%)	0.282	21 (36.8%)	38 (28.4%)	0.322
Smoking	3 (27.3%)	27 (15.0%)	0.277	6 (10.5%)	24 (17.9%)	0.286
Immuncompromised states	0 (3.8%)	6(3.3%)	0.535	3 (5.3%)	5(2.2%)	0.250
Pneumonia	5(45.5%)	88(49.4%)	1.00	27 (47.4%)	66 (50%)	0.740
Laboratory Findings median (Q3-Q1)						
	Myalgia (+) (11)	Myalgia (-) (180)	*P* *(myalgia+/-)*	Headache (+) (57)	Headache (-) (134)	*P’ (headache+/-)*
Leukocyte value at admission(K/ µL)	6200.0 (8730.0-4910.0)	6350.0 (8085.0-4982.5)	NS	6140.0 (7660.0-4860.0)	6615.0 (8420.0-5107.0)	0.281
Lymphocyte value at admission(K/ µL)	2100.0 (2439.0-1880.0)	1827.0 (2511.0.-1190.0)	NS	1820.0 (2315.5.0-1277.0)	1878.0 (2553.8-1185.5)	0.792
Ferritin level (µg/L)	57.1(108.0-19.8)	96.0 (192.5–35.5)	**0.049**	37.0 (119.7–20.0)	117.0 (220 − 50.0)	**< 0.001**
CRP level at admission (mg/L)	5.9 (12.2-2.0)	2.9 (8.8-2)	NS	2.4 (6.4-2.0)	3.3 (13.6-2.0)	0.255
CK at admission (U/L)	57.0 (82.0–45.0)	72.0 (97.5–50.5)	NS	62.0 (85.0-46.5)	75.0 (99.0–52.0)	0.056

CRP, c-reactive protein; CK, creatinine kinase; LDH, lactate dehydrogenase; ALT, alanine aminotransferase; AST, aspartate aminotransferase

Q1, first quartile; Q3, third quartile. Please note that interquartile range is IQR = Q3-Q1.

**Table 4:** Backward stepwise logistic Regression Analysis: Identifying headache variables associated with long COVID headache

Variable	Wald	OR	95% CI		*p*
Female gender	14.781	4.596	2.112	10.001	**< 0.001**
Asthenia	3.751	2.174	0.991	4.771	0.053
Neuropathic pain	7.631	3.038	1.381	6.684	**0.006**

Variable(s) entered on step 1: female sex, cough, asthenia, neuropathic pain, myalgia and ferritin levels at admission

### Myalgia and polyarthralgia

Myalgia was detected in 11 patients, accounting for 5.76% of the total patient population. Additionally, polyarthralgia was observed in 2 patients, representing 1.05% of the total.

The extremity was the most common site of pain in 6 patients (54.5%), followed by back pain in 4 patients (36.4%) and widespread pain in 1 patient (9.1%). The mean intensity of myalgia was 6.3 ± 2.1 (Table 5).

**Table 5:** Characteristics of pain in patients with persistent myalgia

*N* of patient	Localization	Severity At Onset	Severity After 1.5 Years
1	Generalized	7	7
2	Back	8	3
3	Extremity	8	4
4	Back	6	6
5	Extremity	7	4
6	Extremity	9	9
7	Back	8	7
8	Back	7	7
9	Extremity	9	5
10	Extremity	8	9
11	Extremity	9	8

In Table 3, a comparison is presented between patients with and without long-term myalgia. Among the parameters investigated, it was observed that patients with long-term myalgia exhibited a higher incidence of headache, arthralgia, and low ferritin levels compared to patients without a history of long-term myalgia.

Out of the 191 patients, only two (1.05%) reported persistent polyarthralgia. Both of these patients also reported myalgia.

### Neuropathic pain symptom

Persistent neuropathic pain was identified in 8 individuals, which accounts for 4.2% of the total cases.

The intensity of persistent neuropathic pain was 6.9 ± 2.0. All patients reported burning pain localized to extremities, indicative of neuropathic pain. In comparison to other COVID-19 patients, those exhibiting persistent neuropathic pain characteristics were found to be female (*p* = 0.02), older age (*p* = 0.02) and a higher incidence of symptoms as sore throat (*p* = 0.003) and headache (*p* = 0.04). When compared with patients with persistent neuropathic pain to those without persistent neuropathic pain we did not find an association with hospitalization, hospitalization in intensive care unit, fever, cough, anosmia, myalgia, arthralgia, comorbid disease, smoking, immuncompromised state, pneumonia and laboratory findings including leukocyte values, lymphocyte values, ferritin levels, CRP and CK levels at admission (*p* > 0.05). Only 1 patient out of 8 patients was receiving gabapentin for neuropathic pain.

## Discussion

The study aims to determine the frequency and characteristics of persistent pain in individuals diagnosed with COVID-19 and compare them with those who have recovered or did not experience persistent pain. We also aimed to gain a better understanding of the nature and severity of persistent pain in long-term COVID-19 patients and identify the predictors and factors that contribute to its development. According to the findings of this study, after 1.5 years of COVID-19 infection, 31.9% of individuals were still suffering from at least one type of pain symptom, with headache being the most common persistent one.

### Headache

The prevalence of post COVID headache is 29.8%, as the most commonly reported pain symptom. However, the prevalence of new-onset long-COVID headaches stands at 3.66%. It can be particularly challenging to diagnose long COVID headaches in patients with a history of primary headaches. Out of the 57 patients in our study, 7 presented with newly onset headaches, while the others with pre-existing primary headaches described an increase in headache frequency, pain intensity, or a change in headache type. It was difficult to distinguish long COVID headaches in patients with pre-existing primary headaches since headaches such as tension-type headache and migraine are common in the population. Therefore, we specifically assessed these alterations as a long COVID symptom. Consequently, the number of patients identified as having persistent symptoms of COVID-19 in our study may be comparatively higher than in other studies. A previous meta-analysis showed that headache is a long COVID symptom, with a prevalence of 16.5% at 3 months and 8.4% at 6 months [17]. A large population study found that 16% of individuals experienced persistent headaches after 9 months. However, these studies did not evaluate the specific type of prior headache history [18].

Diagnosing long COVID headaches can be challenging, especially for patients with pre-existing primary headaches, such as tension-type headaches and migraines, which are common in the general population. Research suggests that long COVID can either trigger new headaches or worsen existing ones, often mimicking tension-type headaches [19]. Additionally, The International Headache Society classification currently lacks a specific definition for this type of headache, highlighting the need for further research [20]. Notably, a recent study found that individuals with pre-existing migraines were more susceptible to a wider range of long COVID symptoms, including fatigue, anxiety, and chronic headaches, compared to those without migraines [21]. This finding underscores the potential vulnerability of certain patient groups to long COVID complications.

Differentiating secondary headaches in patients with new-onset headaches is another crucial issue. However, due to the phone-based methodology used in our study, we were unable to rule out the possibility of secondary headaches in these patients. On the other hand, none of the participants were suspected of secondary headaches after being questioned.

The underlying mechanisms behind post-COVID pain are uncertain, however, some hypotheses could explain the pathophysiology. One hypothesis suggests that cell-to-cell inflammatory mechanisms induced by SARSCoV-2 (i.e., cytokine and interleukin storms) can lead to hyperexcitability of the peripheral and central nervous systems through various pathways, resulting in the development of de novo post-COVID pain or, in predisposed individuals, a worsening of pre-existing pain symptoms, as reported in this study. Another hypothesis suggests that the SARS-CoV-2 virus can cause an exaggerated immune response by inducing hyperactivation of T cells, macrophages, and natural killer cells. Such a response would promote the facilitation of the central nervous system.

Our research indicates that female patients are more likely to experience long-term headaches. Furthermore, the existence of neuropathic pain seems to be a dependable indicator of long-term headaches in COVID-19 patients. These findings may prove valuable in developing effective treatment strategies and conducting further research on the causes and underlying mechanisms of long-term headaches.

Only 1 patient with a headache frequency exceeding 15 days per month received preventive treatment. This stark discrepancy suggests an underutilization of preventive therapies for headaches, potentially leading to suboptimal patient outcomes and perpetuating the debilitating effects of long COVID.

### Myalgia and polyarthralgia

The prevalence of myalgia 1.5 years after COVID-19 infection in our study was 5.8%, which was 49.7% in the acute phase in the same population.

The prevalence of musculoskeletal pain in long-COVID varies widely in studies ranging from 0.3–65.2% [22–24].

The most common site of myalgia was extremities in our study. Similar to our previous study, we found that individuals who experience persistent headaches and arthralgia are more likely to suffer from persistent myalgia. The precise pathophysiologic mechanisms underlying the strong association between these persistent pain syndromes 1.5 years after COVID-19 infection remain elusive. However, it may be attributed to the generalized inflammation and cytokine response which leads to nociceptive activation and central sensitization, resulting in pain syndromes.

Moreover, our findings suggest that the presence of persistent myalgia in patients with COVID-19 is not associated with pneumonia, hospitalization, fever, cough, fatigue, and comorbid diseases. This indicates that persistent myalgia may not be a result of a severe COVID-19 infection. There are limited studies on specific features of musculoskeletal pain in long COVID patients.

According to a recent nationwide study, 5.3% of non-hospitalized COVID-19 survivors experienced widespread pain one year after COVID-19 infection. The study identified several risk factors associated with the development of this pain, including older age, higher body mass index, female sex, history of migraine, neurological disorders, stress, whiplash, type-2 diabetes mellitus, and lower socioeconomic status [13].

### Neuropathic pain symptom

Our findings showed that 4.2% of COVID-19 patients have persistent neuropathic pain symptoms after 1.5 years and gender, age, presence of headache, and sore throat may be contributing factors to the development of persistent neuropathic pain in COVID-19 patients. In contrast to myalgia, there is limited data available for neuropathic pain.

While studies have been conducted to investigate neuropathic pain during the acute phase of COVID-19 infection, the frequency and characteristics of this pain in the long term have only been investigated in a limited number of studies [25, 26]. A study showed that 24.6% of new COVID-19 patients report chronic neuropathic pain after 6 months of infection [27]. According to another study, the percentage of persistent neuropathic pain was found to be 7.6% after 8 months [28].

The observed variances may be ascribed to the gradual amelioration of neuropathic pain symptoms manifested by patients over time. Our previous study found that 25% of patients experienced neuropathic pain during the acute phase. Reduction in neuropathic pain may be due to treatment or lifestyle changes as well as relieving pathogenic mechanisms.

Only 1 out of 8 patients with neuropathic pain was receiving medication. This finding suggests a potential gap between the identification of neuropathic pain and its treatment in long COVID.

### Limitations and strengths of the study

It is important to note that our study has some limitations. Firstly, the information we gathered was based on self-reported symptoms during telephone calls, which may be influenced by recall bias and subjective interpretation by the participants. Furthermore, the use of a semi-structured and unvalidated questionnaire may limit the generalizability of our findings to other populations or settings. Secondly, the risk of developing new-onset pain may be significantly influenced by previous pain conditions. Our study primarily included mildly and moderately infected COVID-19 patients, potentially limiting the generalizability of our findings to the broader population. Additionally, healthcare disparities, geographic variations, and potential ethnic variations within the sample might further restrict the applicability of our results to the entire COVID-19 population. Lastly, we did not have a control group, making it challenging to distinguish between symptoms associated with long COVID and those that might occur naturally in the general population.

Our study had the advantage of being able to assess pain symptoms over an extended period of time, allowing us to gain a deeper understanding of how these symptoms develop and change over time. By evaluating pain symptoms over time, we identified patterns and trends that weren’t visible in shorter studies. These findings can be valuable in developing effective treatment strategies and improving patient outcomes.

In conclusion, our study examines the frequency and predictors of persistent pain symptoms among COVID-19 patients after 1.5 years. The findings suggest that some patients may experience an increase in the frequency and intensity of pre-existing headaches, while others may develop new onset headaches or experience a transition in the type of headache they have already been suffering. Additionally, some patients continue to experience other types of pain such as myalgia, arthralgia, or neuropathic pain after 1.5 years of COVID infection. These pain syndromes appear to be strongly interconnected, indicating a shared pathway.

### Supplementary Information

Below is the link to the electronic supplementary material.

Supplementary file1 (DOCX 19 KB)
