# Supplementary material for: Beyond the acute: pain in long COVID survivors at 1.5 years
Source: Neurol Sci. 2024 May 31;45(9):4109–17. doi: 10.1007/s10072-024-07620-7 (PMC11306299; doi:10.1007/s10072-024-07620-7)
Supplement: Supplementary file 1 — Supplementary file1 (DOCX 19 KB) [file 10072_2024_7620_MOESM1_ESM.docx]

**Name:**

**Gender:**

**Patient Number:**

**Before we begin this questionnaire, I want to inform you that this is a follow-up study to a previous survey you participated in 1.5 years ago. The purpose of this questionnaire is to assess your experience with post-COVID-19 symptoms, specifically headaches, muscle pain, joint pain, and neuropathic pain.** **Your participation in this study is entirely voluntary. You may choose to withdraw from the questionnaire at any time.** **The questionnaire will take approximately 20-25 minutes to complete. Do you understand and agree to participate in this follow-up study?**

**Yes No**

**HEADACHE**

1. **Do you still have headaches after being diagnosed with COVID-19?**

Yes No

**If yes;**

1. **What is the frequency of your headaches after COVID-19 infection? (last 1 month)**

<1 per month 1-4 per month 5-14 per month ≥15 per month

1. **How has the frequency of your headaches changed after COVID-19 infection compared to your pre-COVID-19 baseline?**

Increased

Decreased

Same

1. **How severe is your headache after COVID-19 infection?**

Mild

Moderate

Severe

Very severe

1. **How has the severity of your headaches changed after COVID-19 infection compared to your pre-COVID-19 baseline?**

Increased

Decreased

Same

1. **Headache type (there may be more than 1 answer)**

Pulsating

Pressing

Fiery

Stabbing

Other

1. **Where is the localization of your headache? (there can be more than one answer)**

Single-sided

Double-sided

Double sided significant on one side

1. **Which symptoms do you have accompanying your headache (there may be more than one answer)**

Nasal congestion

Postnasal drip

Anosmia

Fever

Osmophobia

Nausea

Vomiting

Photophobia

Phonophobia

Increase by movement

1. **Did you have any other neurological complaints accompanying the headache? (there can be more than one answer**)

Double vision

Visual disorder

Speech disorder

Dizziness

Numbness/weakness on one side of body

Other

1. **Are you currently taking any pain relievers for headache? If so, please specify which ones you have taken.**

YES NO

Simple analgesics

NSAID

Combined analgesics

Opioids and derivatives

Others

1. **Are you on prophylactic treatment? If yes; which prophylactic treatment have you taken for your headache?**

YES NO

…………….

**MYALGIA**

1. **Are you still experiencing muscle pain that started after the COVID-19 infection?**

YES NO

**If yes;**

1. **Where is the localization of your muscle pain (myalgia)?**

Widespread

Back/low back pain

Upper and lower limbs

1. **How has the severity of your myalgia changed after COVID-19 infection compared to your pre-covid-19 baseline?**

Increased

Decreased

Same

1. **The severity of your muscle pain**

0 – 10 – 20 – 30 – 40 – 50 – 60 – 70 – 80 – 90 – 100 (the most severe pain imaginable)

1. **Are you currently taking any pain relievers for muscle pain? If so, please specify which ones you have taken.**

YES NO

Simple analgesics

NSAID

Combined analgesics

Opioids and derivatives

Others

1. **Are you on prophylactic treatment? If yes; which prophylactic treatment have you taken for your muscle pain?**

YES NO

…………….

**ARTHRALGIA**

1. **Are you still experiencing generalized joint pain that started after the COVID-19 infection?**

YES NO

**If yes;**

1. **How has the severity of your joint pain changed after COVID-19 infection compared to your pre-COVID-19 baseline?**

Increased

Decreased

Same

1. **What is the severity of your joint pain?**

0 – 10 – 20 – 30 – 40 – 50 – 60 – 70 – 80 – 90 – 100 (the most severe pain imaginable

1. **Are you currently taking any pain relievers for joint pain? If so, please specify which ones you have taken.**

YES NO

Simple analgesics

NSAID

Combined analgesics

Opioids and derivatives

Others

**NEUROPATHIC PAIN**

1. **Are you still experiencing neuropathic pain symptoms like burning, tingling, or shooting sensations that started after the COVID-19 infection?**

YES NO

**NEUROPATHIC PAIN QUESTIONNAIRE**

1. **Burning pain**

0 – 10 – 20 – 30 – 40 – 50 – 60 – 70 – 80 – 90 – 100 (the most severe pain imaginable)

1. **Overly sensitivity to touch**

0 – 10 – 20 – 30 – 40 – 50 – 60 – 70 – 80 – 90 – 100

1. **Shooting pain**

0 – 10 – 20 – 30 – 40 – 50 – 60 – 70 – 80 – 90 – 100

1. **Numbness**

0 – 10 – 20 – 30 – 40 – 50 – 60 – 70 – 80 – 90 – 100

1. **Electric pain**

0 – 10 – 20 – 30 – 40 – 50 – 60 – 70 – 80 – 90 – 100

1. **Tingling pain**

0 – 10 – 20 – 30 – 40 – 50 – 60 – 70 – 80 – 90 – 100

1. **Squeezing pain**

0 – 10 – 20 – 30 – 40 – 50 – 60 – 70 – 80 – 90 – 100

1. **Pain in the form of freezing**

0 – 10 – 20 – 30 – 40 – 50 – 60 – 70 – 80 – 90 – 100

1. **How unpleasant is your usual pain?**

0 – 10 – 20 – 30 – 40 – 50 – 60 – 70 – 80 – 90 – 100 (the most unpleasant pain imaginable)

1. **How overwhelming is your usual pain**

0 – 10 – 20 – 30 – 40 – 50 – 60 – 70 – 80 – 90 – 100 (the most overwhelming pain imaginable)

1. **Increased pain due to touch**

0 – 10 – 20 – 30 – 40 – 50 – 60 – 70 – 80 – 90 – 100 (the maximum increase imaginable)

1. **Increased pain due to weather changes with change of air**

0 – 10 – 20 – 30 – 40 – 50 – 60 – 70 – 80 – 90 – 100 (the biggest increase imaginable)

1. **Where is your pain with neuropathic characteristics?**

Upper and lower limbs

Back/low back

Neck

Widespread

1. **How has the severity of these neuropathic pain symptoms changed after COVID-19 infection compared to your pre-COVID-19 baseline?**

Increased

Decreased

Same

1. **Are you currently taking any pain relievers for neuropathic pain symptom If so, please specify which ones you have taken.**

YES NO

Tricyclic antidepressants

Selective serotonin reuptake inhibitors/Serotonin-norepinephrine reuptake inhibitors

Gabapentin/pregabalin

Carbamazepine

Analgesics (opioids, NSAID, paracetamol)

Others
